# A Mahalanobis Surrogate-Assisted Ant Lion Optimization and Its Application in 3D Coverage of Wireless Sensor Networks

**DOI:** 10.3390/e24050586

**Published:** 2022-04-22

**Authors:** Zhi Li, Shu-Chuan Chu, Jeng-Shyang Pan, Pei Hu, Xingsi Xue

**Affiliations:** 1 College of Computer Science and Engineering, Shandong University of Science and Technology, Qingdao 266590, China; lizhi@sdust.edu.cn (Z.L.); jspan@cc.kuas.edu.tw (J.-S.P.); hupei@nyist.edu.cn (P.H.); 2 Department of Information Management, Chaoyang University of Technology, Taichung 41349, Taiwan; 3 Fujian Provincial Key Laboratory of Big Data Mining and Applications, Fujian University of Technology, Fuzhou 350118, China; xxs@fjut.edu.cn

**Keywords:** ant lion optimization, surrogate model, mahalanobis distance, radial basis function network, 3D coverage, wireless sensor networks

## Abstract

Metaheuristic algorithms are widely employed in modern engineering applications because they do not need to have the ability to study the objective function’s features. However, these algorithms may spend minutes to hours or even days to acquire one solution. This paper presents a novel efficient Mahalanobis sampling surrogate model assisting Ant Lion optimization algorithm to address this problem. For expensive calculation problems, the optimization effect goes even further by using MSAALO. This model includes three surrogate models: the global model, Mahalanobis sampling surrogate model, and local surrogate model. Mahalanobis distance can also exclude the interference correlations of variables. In the Mahalanobis distance sampling model, the distance between each ant and the others could be calculated. Additionally, the algorithm sorts the average length of all ants. Then, the algorithm selects some samples to train the model from these Mahalanobis distance samples. Seven benchmark functions with various characteristics are chosen to testify to the effectiveness of this algorithm. The validation results of seven benchmark functions demonstrate that the algorithm is more competitive than other algorithms. The simulation results based on different radii and nodes show that MSAALO improves the average coverage by 2.122% and 1.718%, respectively.

## 1. Introduction

Metaheuristic algorithms such as the Artificial Bee Colony (ABC) [1], Gray Wolf Optimizer (GWO) [2], Cat Swarm Optimization (CSO) [3], Differential Evolution (DE) [4], Ant Lion Optimization (ALO) [5], Memetic Algorithm (MA) [6] and Particle Swarm Optimization (PSO) [7] are widely employed in modern engineering applications [8,9]. Metaheuristic algorithms have the advantage of not needing to study the features of the objective function such as convex, linear and others. Therefore, these algorithms have good performance in some engineering areas. These fields include structural optimization design of truss topology [10], traveling salesman problem [11], reliability optimization of complex systems [12], feature selection [13], vehicle routing problem [14], wireless sensor networks [15,16] and others. However, they have some common characteristics in some high-dimensional and high-level application scenarios, such as time-consuming evaluation, which may take minutes to hours or even days to acquire one solution. In the past decades, a great number of surrogate models assisting metaheuristic algorithms have emerged to address this problem. For example, there are Kriging [17], Gaussian process(GP) [18,19], polynomial regression(PR) [20,21], support vector machines(SVM) [22], artificial neural network(ANN) [23,24] and radial basis function network (RBFN) [25,26]. An increasing number of comparative experiments have been conducted on the quality of various models with various fitness functions. The results demonstrate that the radial basis function network performs superior with the high dimension of complex optimization problems on smaller training data [27]. GP [28] is appropriate for modeling complex problems in the global field. When suffering from the problem of searching for the best hyperparameters, GP has some disadvantages such as being time-consuming, which is a major disadvantage to using GP.

An increasing number of surrogate models have emerged in the current research field to settle these expensive calculation problems. Liu et al. [29] employed a Gaussian process to construct a model which solves the high-dimension computationally optimization problems through dimension reduction techniques. In [30], Regis et al. used an RBF global surrogate to assist particle swarm optimization. They first proposed multiple trial solutions for each particle. This model selects the promising particle which belongs to one of many trial particles. Zhou et al. [31] presented a new surrogate model framework that is employed to solve expensive computational problems. It utilizes a variety of hierarchical models to replace the real fitness function in order to save computing resources. RBFN is used to construct the model, which uses the Gaussian process. A trust-region basing gradient search is used to build the local model. Using a neural network, Jin et al. [24] constructed a global surrogate model that assists a covariance matrix adaptation strategy and generation control. Praveen et al. [32] built a global surrogate model assisting particle swarm algorithm by using radial basis function. In the process of expensive fitness computation, this model selects the most promising particle in all screened particles. A support vector regression (SVR) model was utilized by Wang et al. [33] to assist a multi-objective evolutionary algorithm to search for the best baseline sequence and resource distribution solution. This algorithm saves completion time and resource costs. In [34], Chugh et al. presented a Kriging model which selects one method to reduce the calculation time without damaging the fitness accuracy. This algorithm effectively achieves a balance between exploration and exploitation.

In recent times, the surrogate models have been divided into two class models involving the global and local models. In [35], Ong et al. presented one model which can use alternately real fitness functions and constraint functions via the trust region approach. The constraint function is a surrogate model which can be computed more cheaply. Sun et al. [36] proposed a novel fitness function method for PSO. They call the method FESPSO, and it reduces a lot of time in estimating particles with real fitness functions. Lim et al. presented one algorithm using various surrogate models assisting evolutionary algorithms in the local search range of the memetic algorithm [37]. The complex surrogate model is employed to produce reliable and precise fitness values. It uses ensemble and smoothing models to search simultaneously. However, the metaheuristic algorithm effectiveness relies not only on the selection of the surrogate model, but also on the preference of the training sample set. The global and the local surrogate models need to select samples to train the model, so the selection of samples is crucial to the model’s training. Therefore, we present a new distance sampling method for training models.

In this article, we propose a novel efficient Mahalanobis sampling surrogate model assisting Ant Lion Optimization [5] algorithm where the optimization effect be upgraded for expensive calculation problems. This model includes three surrogate models—the global model, local surrogate model and Mahalanobis sampling surrogate model. Mahalanobis distance also excludes the interference of correlations between variables. In the Mahalanobis distance sampling model, we calculate the average Mahalanobis distance between each ant and others. Then, the algorithm sorts these average distances from small to large of all ants in order to obtain the top gs ants. The sampling data from these three models can be stored in one database called DB. The surrogate assisting ALO is applied to the 3D coverage in wireless sensor networks. The experimental data show that the presented algorithm MSAALO is efficient in addressing expensive calculation problems.

The remainder of this article is organized as follows. In Section 2, this paper briefly reviews the related knowledge, including the ALO algorithm, the radial basis function network, the coverage model of wireless sensor networks and Mahalanobis Distance. In Section 3, the article introduces MSAALO and its application in the node coverage in WSN in detail. The experimental results with the other three algorithms in seven benchmark functions and the simulation application results in the 3D coverage of WSN are revealed in Section 4. In Section 5, the article concludes the proposed MSAALO and its future work.

## 2. Related Work

### 2.1. Ant Lion OPtimization

From the past decades to the present, many researchers optimized metaheuristic algorithms [38,39,40] in order to apply them in engineering fields [41,42,43,44,45]. The Ant Lion Optimizer is an original metaheuristic algorithm which was introduced by Mirjalili in 2015 [5]. An ant lion moves along a circular hollow made of sand and catches ants using their massive jaw. When ants randomly move into holes, antlions will seize ants, rebuild their hollows and wait for another ant. The ALO algorithm mainly includes two members: ant and antlion. The member with the optimal fitness value among the antlions is selected as the elite antlion. The execution process of the algorithm mainly includes two processes, stochastic walking of ants and position movement of antlions. The moving track can be influenced by traps. The antlion with a high proportion has a higher likelihood of seizing ants. When the ants have better fitness than the antlion, the antlion needs to relocate its position to build a new trap. During the random walk of ants, the trajectory of the ants is modeled according to the following Equation (1):

(1) $$X\left(t\right)=[0,cumsum\left(2r\left(t_{1}\right)-1\right);cumsum\left(2r\left(t_{2}\right)-1\right);...;cumsum\left(2r\left(t_{T}\right)-1\right);]$$

where cumsum is equal to the cumulative sum; *t* is the current step; *T* is the maximum amount of rounds. $r(t_{i})$ is a stochastic function. It can be defined as Equation (2).

(2) $$r\left(t_{i}\right)=\left\{\begin{array}{ccc} 1 & \; & if\;rand\_num>0.5\\ 0 & \; & if\;rand\_num\leq 0.5 \end{array}\right.$$

where rand_num is a random number which is generated with uniform distribution in the interval [0, 1] and 1≤i≤T. *T* random values are generated during each iteration. Each ant employs Equation (3) to normalize its position in order to prevent ants from going out of the search space. The equation can be expressed as follows.

(3) $$X_{i}^{t}=\frac{\left(X_{i}^{t}-d_{i}\right)\left(b_{i}^{t}-a_{i}^{t}\right)}{c_{i}-d_{i}}+b_{i}^{t}$$

where $d_{i}^{t}$ is the upper restraint of the random walk of *i*-th variable, $d_{i}$ is the lower restraint of random walk in *i*-th variable, $a_{i}^{t}$ is the minimum value of the *t*-th iteration for the *i*-th dimension. $c_{i}$ is the maximum value for the *i*-th dimension. The definitions of $b_{i}^{t}$ and $a_{i}^{t}$ are expressed as follows.

(4) $$a_{i}^{t}=Antlion_{i}^{t}+a^{t}$$

(5) $$b_{i}^{t}=Antlion_{i}^{t}-b^{t}$$

where $a^{t}$ is the minimum value of all variables at the *t*-th iteration; $b^{t}$ is the maximum value of all variables at the *t*-th iteration. Each ant can only be preyed upon by one antlion via Roulette Strategy [46]. The antlion with a higher fitness value is more likely to capture the ant. In addition, the antlion casts sand at the edge of the trap to keep the ant from running away if an ant falls into a trap made by an antlion. At this point, the range of ants randomly wandering will be drastically reduced. The following Equations (6) and (7) simulate this capture process.

(6) $$a^{t}=\frac{c^{t}}{I}$$

(7) $$b^{t}=\frac{d^{t}}{I}$$

where *I* is the ratio factor which can be defined as follows.

(8) $$I=\left\{\begin{array}{ccc} 1 & \; & if\;g\leq 0.1G\\ 10^{\omega }*\frac{g}{G} & \; & if\;g>0.1G \end{array}\right.$$

where *g* is the current round, *G* is the maximum number of iterations. ω can be fetched via the following Equation (9).

(9) $$\omega =\left\{\begin{array}{lll} 1, & \; & if\;0<g\leq 0.1G\\ 2, & \; & if\;0.1G<g\leq 0.5G\\ 3, & \; & if\;0.5G<g\leq 0.75G\\ 4, & \; & if\;0.75G<g\leq 0.9G\\ 5, & \; & if\;0.9G<g\leq 0.95G\\ 6 & \; & if\;0.95G<g\leq G \end{array}\right.$$

where ω is used to control the degree of exploration, and varies with the number of iterations. ω controls the balance between convergence and exploration of the algorithm. The convergence ability of ALO increases in the later generation. Therefore, ω grows with the number of iterations.

### 2.2. Radial Basis Function Network

As a sort of artificial neural network [13], Radial Basis Function Network was introduced for the first time by Hardy [47] in order to fit high-dimensional nonlinear data. Furthermore, RBFN [48] has the ability to perform well in global and local modeling. This paper employs RBFN assisting Ant Lion Optimization to obtain models. The RBFN is engaged in this work as follows.

(10) $$\hat{y}\left(x\right)=\sum_{i=1}^{N}\alpha_{i}\varphi (∥x-x_{i}∥)$$

where $∥\cdot ∥,\alpha_{i}$ and φ are the Euclidian model, the weight coefficients which can be acquired via the linear system Equation (11) and the kernel function. *x* is the center point; $x_{i}$ is the *i*-th sample. Typical RBF kernels include multiquadric splines, linear splines, Gaussian function, cubic splines and thin-plate splines. This research utilizes a Gaussian kernel to build a local model and exploit linear splines to construct a global model because of their different properties. The form of the Gaussian kernel function is shown in Equation (12).

(11) $$\alpha =\phi^{-1}ϝ$$

(12) $$\varphi \left(x\right)=exp\left(\frac{-x^{2}}{\beta }\right)$$

where $\phi =[\varphi (∥x_{i}-x_{j}{∥)]}_{M\times M}$ is the kernel matrix. When only the matrix $X={\left[x_{1},x_{2},...,x_{M}\right]}^{T}$ is different, the ϕ is a positive definite. In accordance with the Gaussian kernel function’s shape parameter, β is equivalent to $D_{max}{\left(dM\right)}^{-1/d}$ where $D_{max}$ is the maximum distance among the training set. In the data set of RBFN, the location and fitness values of the $i_{th}$ member are $x_{i}=\left(x_{i}^{1},x_{i}^{2},...,x_{i}^{D}\right)\epsilon R^{D}$ and $f(x_{i})$, respectively.

### 2.3. Mahalanobis Distance

Mahalanobis Distance [49,50] is a measure of distance, which can be regarded as a correction of Euclidean distance. It corrects the problem of the scales of various dimensions in Euclidean distance being inconsistent and related. The distance of individual data points is expressed by Equation (13). The Mahalanobis distance between data points *x* and *y* can be computed by Equation (14).

(13) $$D_{P}\left(y\right)=\sqrt{{\left(y-\mu \right)}^{T}\sum^{-1}\left(y-\mu \right)}$$

(14) $$D_{P}\left(y,z\right)=\sqrt{{\left(y-z\right)}^{T}\sum^{-1}\left(y-z\right)}$$

where ∑ is the covariance matrix of multidimensional random variables; μ is the sample’s mean. When the covariance matrix is a unit vector means, the dimensions are independent and identically distributed, and the Mahalanobis distance becomes Euclidean distance.

### 2.4. The Coverage Model of Wireless Sensor Networks

The WSN [51] have become more and more popular in the research field because of their significant value in real-world applications. For instance, seismic detection, intelligent home and health care applications apply wireless sensor networks. The coverage ability of nodes affects the lifetime and performance of the whole WSN. There are two classical sensor detection models, the 0–1 model and the probability model. In this work, we will optimize the WSN coverage using the 0–1 model. In the 0–1 model, the sensing radius is set to *r*. If the Euclidean distance of the node *P* and the target objection *C* is less than the radius r, the probability of the model is equal to 1. On the contrary, the probability is 0. It can be expressed by the following Equation (15).

(15) $$F\left(P,C\right)=\left\{\begin{array}{l} 1\;if\;D(P,C)\leq r\;\&\&\;no\;obstacle\\ 0\;if\;D(P,C)>r \end{array}\right.$$

## 3. Mahalanobis Surrogate-Assisted Ant Lion Optimization and Node Coverage in Wireless Sensor Network

### 3.1. Mahalanobis Surrogate-Assisted Ant Lion Optimization

A new surrogate model assisting the Ant Lion optimization is proposed in this part. This surrogate-assisted antlion algorithm includes three layers of surrogate models which are the global surrogate, one sampling surrogate using Mahalanobis distance and the local surrogate model, respectively. A sample database is employed to store the ants evaluated using the real fitness function. Every sample includes its position and fitness value. Furthermore, the global surrogate model can perform global fitting of smooth functions. The novel model is trained by the first gs samples of the database. This algorithm integrates the local surrogate model and the Mahalanobis sampling model with the global surrogate model. Then, we can effectively increase the execution efficiency of the Ant Lion algorithm. An increasing number of samples will multiply the time complexity and space complexity of the construction of the surrogate model. Therefore, the first gs samples in the database are elected to form the agent model. Operating the global surrogate model, MSAALO selects the ants with the better fitness value in each generation of the algorithm and stores them in the sample database. In each iteration, all ants are evaluated with the local agent model and compared with the fitness values of the antlion. Then, the ants with better fitness that do not belong to the previous generation of antlion populations are evaluated using the true fitness evaluation function and deposited into the sample database. Algorithm 1 shows the execution process of MSAALO. DB is the samples database.

Independent of the dimensions, the Mahalanobis distance of the two positions is independent of the measurement for the original data in units. The Mahalanobis distance between two ants is computed from normalized data. Hence, we use Mahalanobis distance to sample. The Mahalanobis distance eliminates the interference correlation of variables as well. In the Mahalanobis distance sampling model, we calculate the distances between each ant and others. We compute the average distance of each ant, and then the algorithm sorts these average distances from small to large in order to get the top gs ants. If the covariance matrix is not full of rank, this algorithm uses Euclidean distance to select samples. The computation Equation (16) of ant $x_{i}$ can be expressed as follows.

(16) $$Average\left(x_{i}\right)=\frac{1}{N}\sum_{j=1}^{n}F_{D}\left(x_{i},x_{j}\right)$$

where $F_{D}\left(x_{i},x_{j}\right)$ can be computed through Equation (14).
**Algorithm 1** Surrogate-assisted Ant Lion optimization algorithm.**Input:** The lower boundary lb, the upper boundary ub, the dimension dim, the size of population N, the maximum iteration Maxiteration, the objective function fobj;1:**Initialization:** Generate initial samples using Latin hypercube sampling, evaluate the fitness values of initial samples using the expensive real fitness function and save them into the DB;2:i = 1;3:**while**i≤Maxiteration**do**4:   Update DB by using the Global surrogate model;5:   Update DB by using the Mahalanobis distance surrogate model to sample;6:   Update DB by using the Local surrogate model;7:**end while****Output:** The best fitness value and its position.

Algorithm 2 introduces the execution process of the local model. The number of samples that have been utilized for training the model in the DB is gs. The local surrogate model mainly acquires the function model around the currently best ant to improve the search ability of the algorithm. Using the real fitness function to evaluate, MSAALO selects the ant smaller than the current antlion. DB will be updated by these ants.
**Algorithm 2** Local surrogate model.**Input:** Archive DB;1:Select top gs samples from the sorted DB database;2:The Local surrogate model ($F_{l}$) can be trained by these gs samples;3:Generate the population by ALO algorithm;4:Estimate the fitness value of each ant in the colony by local surrogate model;5:**if** the fitness value of ants are better than antlions **then**6:   Evaluate the fitness value of these ants using the real fitness function and store them into DB;7:**end if**8:Update Elite antlion and antlions;**Output:** The updated archive DB, the Elite antlion vector E and its fitness value $F_{l}\left(E\right)$.

Algorithm 3 outlines the specific execution process of the global surrogate model. The global surrogate model improves the exploration ability of the algorithm. In Algorithm 3, the position matrix of all ants is initialized, and the top P best ants are selected as antlions. E and maxgen are the elite antlion position and the maximum amount of iteration of evolution, respectively. N refers to the amount of ants in each generation. $F_{g}\left(x_{i}\right)$ represents the approximation of the *i*-th ant evaluated using the global surrogate model.
**Algorithm 3** Global surrogate model.**Input:** Archive DB, ants position **X**, antlions position matrix **P**, Elite antlion vector **E**;1:Let maxgen = 500;2:Select top P samples from the sorted DB database;3:The Global surrogate model ($F_{g}$) can be trained by these gs samples;4:$F_{g}$ replaces the real fitness function to evaluate the fitness value of ants;5:**while**j≤maxgen**do**6:   **for** i = 1:N **do**7:     Select the first N ants as antlions from the colony;8:     An antlion RA should be selected in accordance with roulette principles;9:     The ants randomly roam around the elite antlion and RA;10:   **end for**11:   **for** i = 1:N **do**12:     $F_{g}$ are employed to assess the fitness value of each ant and stored in the population database colony;13:   **end for**14:   **if** there exists at least an ant that $F_{g}\left(x_{i}\right)$ < $F_{g}\left(E\right)$ **then**15:     E is replaced by an ant position with the best fitness value;16:   **end if**17:**end while**18:Update DB via saving E into DB;**Output:** The updated archive DB, the Elite antlion vector E and its fitness value $F_{g}\left(E\right)$.

### 3.2. The Node Coverage in WSN

In the research field of 2D plane area coverage, an increasing number of methods have emerged. This paper mainly overcomes the challenge of covering the area in 3D with a fixed number of nodes. For the purpose of simulating the real coverage problem, we place these nodes on 3D terrain. In the 3D coverage of WSN, there are many intelligent computation ways to address this 3D problem [51]. This paper optimizes the performance by applying the surrogate model. The 3D coverage problem of WSN are settled using this algorithm.

In the 3D problem, spherical space is obtained when there are two coordinates and radius r. The sphere space is the sensing detection area. Furthermore, we improve the coverage ability of WSN through using MSAALO to optimize the two coordinates. Each ant is equivalent to one deployment strategy. The form (17) of each ant can be expressed as follows:

(17) $$\left[Ant_{1}^{1},Ant_{1}^{2},Ant_{2}^{1},Ant_{2}^{2},.....,Ant_{i}^{1},Ant_{i}^{2},.....,Ant_{n}^{1},Ant_{n}^{2}\right]$$

where *i* refers to the *i*-th node, *n* is the total number of sensor nodes. $Ant_{i}^{1}$ is the first dimension value of the *i*-th node. $Ant_{i}^{2}$ is the second dimension value of the *i*-th node. The coverage rate at the *i*-th round can be computed by the following Equation (18).

(18) $$rate\left(j\right)=\frac{1}{A}\sum_{a=1}^{A}\left(\sum_{b=1}^{B}F\left(P_{b},C_{a}\right)\right)$$

where *A* represents the number of pixels (target objects) of the 3-D terrain. *B* refers to the amount of sensor nodes. $F(P_{b},C_{a})$ indicates whether the a pixel is covered by the b node. It can be calculated by Equation (15).

## 4. Experimental Results

In order to prove the efficiency and effectiveness of MSAALO on time-consuming optimization problems, seven benchmark functions with different characteristics are selected. These test methods consist of unimodal functions, multimodal functions and extraordinary complex multimodal functions. Table 1 shows the detailed characteristics of these benchmark functions. In addition, Yu et al. [52,53], Sun et al. [27] and Li et al. [54] also used these functions to evaluate their proposed algorithms for expensive optimization problems. This research compared MSAALO and the original ALO with the famous PSO and QUATRE algorithms in 30, 50 and 100 dimensions, respectively. All algorithms compared conduct 20 times independently in MATLAB2019b on a computer with an AMD Ryzen 7 5800 H with Radeon Graphics 3.20 GHz processor and 16.0 GB of RAM under the Windows 10 operating system. These comparison results are then analyzed. The surrogate model is constructed using the ’newrb’ function offered in the matlab toolbox.

### 4.1. Parameter Settings

In the experiment, for 30-dimensional and 50-dimensional problems, the population size of MSAALO, PSO, QUATRE, and ALO is set to 100, and the training sample size of MSAALO is set to 50. For a 100-dimensional problem, all comparison algorithms’ population sizes are set to 200, MSAALO training sample size is equal to 150, and the maximum amount of true test function evaluations is equivalent to 1000. For the PSO, the inertia coefficient is equivalent to social cognitive parameter. They are equal to 2.05. The inertia coefficient of PSO decreases linearly. The minimum and maximum inertia parameters are 0.4 and 0.9, respectively. These factors are set the same as those of the particle swarm algorithm [7]. For the QUATRE, c is set to 0.7 following [56]. According to [5], the parameter settings of ALO are the same, and the walking and selection strategy adopts the design used previously in the literature [5]. Latin hypercube sampling is used before evolution. During the population search process of the local surrogate, it will be terminated after twenty consecutive times without further advancement. Each optimization progress is less than the power of $10^{-6}$.

### 4.2. Experimental Analysis on 30- and 50-Dimensional Problems

Table 2 and Table 3 show the experimental results of all algorithms. The mean value, best fitness value, standard deviation, worst fitness value and the Wilcoxon rank-sum test results on the confidence level of 0.05 are presented in the tables. In these tables, ‘+’ represents that MSAALO is better than other algorithms; ‘≈’ shows that MSAALO is no different from other algorithms in terms of the statistical results. PSO is an effective metaheuristic algorithm for optimization problems in engineering fields. QUATRE is a QUasi-Affine TRansformation Evolutionary algorithm [56] which automates the generation of cross matrices.

**Figure 1:**
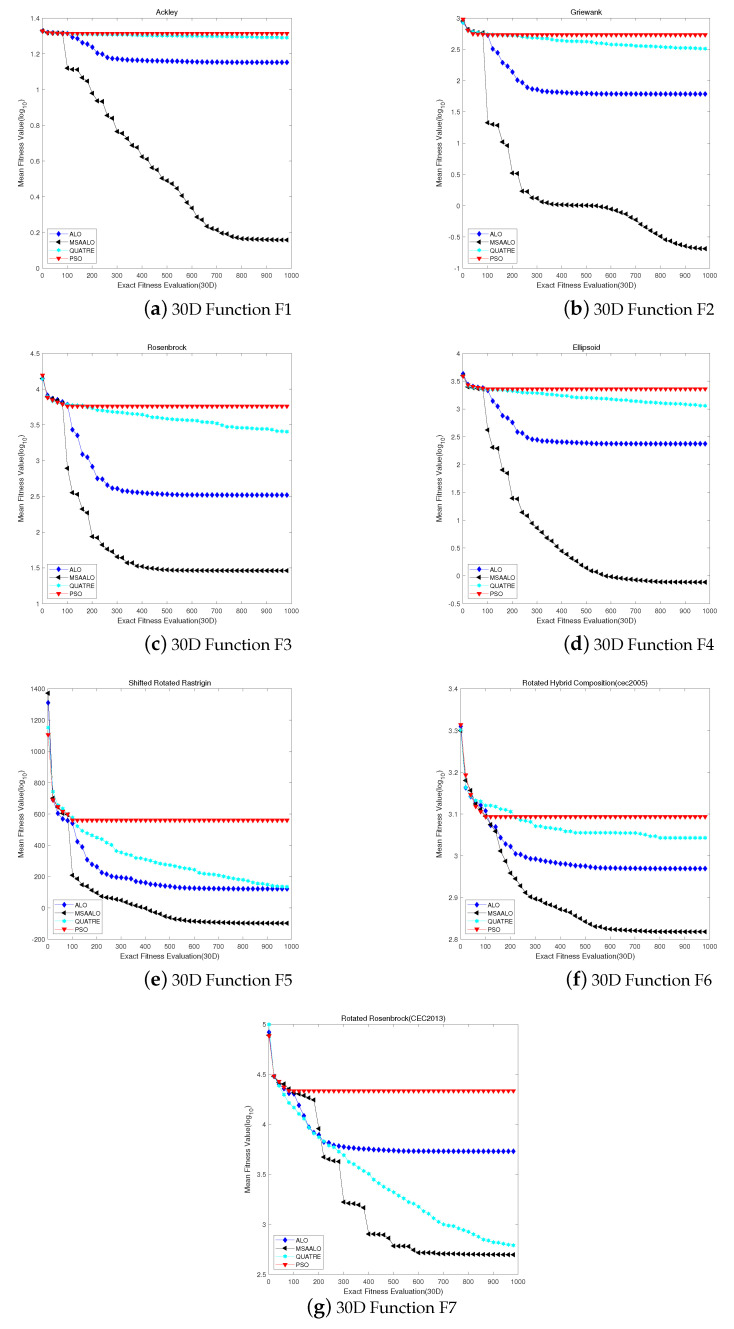
Convergence profiles of algorithms MSAALO, PSO, ALO and QUATRE on 30D with 1000 expensive fitness evaluations.

For the 30-dimensional problems, we conclude that the results of MSAALO are significantly better than the other competition algorithms in the Table 2. Figure 1 shows the converge curve of comparison data. Under the influence of the initializing hypercube sampling, these algorithms have no apparent difference in the early evolution. However, there is the superiority of MSAALO during the later evolution. Compared with the QUATRE algorithm, MSAALO can converge to the best fitness value earlier. Furthermore, MSAALO has an optimum fitness value compared with the QUATRE algorithm. MSAALO converges the best fitness value in 700 calculations approximately, which refers to the computation of the real fitness function. However, QUATRE can reach the optimum fitness value after the maximum calculations or so.

Based on F1–F2 and F4 benchmark function tests, the MSAALO algorithm performs better than the other three algorithms. Compared to the other three algorithms, MSAALO finds better solutions at the beginning of the algorithm. However, the other three algorithms encounter local convergence problems after 300 accurate evaluations. These data prove that MSAALO has an excellent ability to capture the global optimum. From the convergence curves of F1 and F2, MSAALO faces local convergence problems after 900 actual evaluations. For the F3 and F5-F6 benchmark functions, MSAALO decreases faster than the other algorithms, indicating that MSAALO has a more robust global search capability than the other algorithms. Although QUATRE stuck in a local optimum in late stages, the best solution is worse than MSAALO. For the Rotated Rosenbrock Function (F7), MSAALO does not perform as well as QUATRE, but it is better than the other two algorithms. We see that MSAALO performs better than other algorithms in finding the best solutions from Table 2. These examinations indicate that MSAALO achieves a better balance between exploration and exploitation.

**Figure 2:**
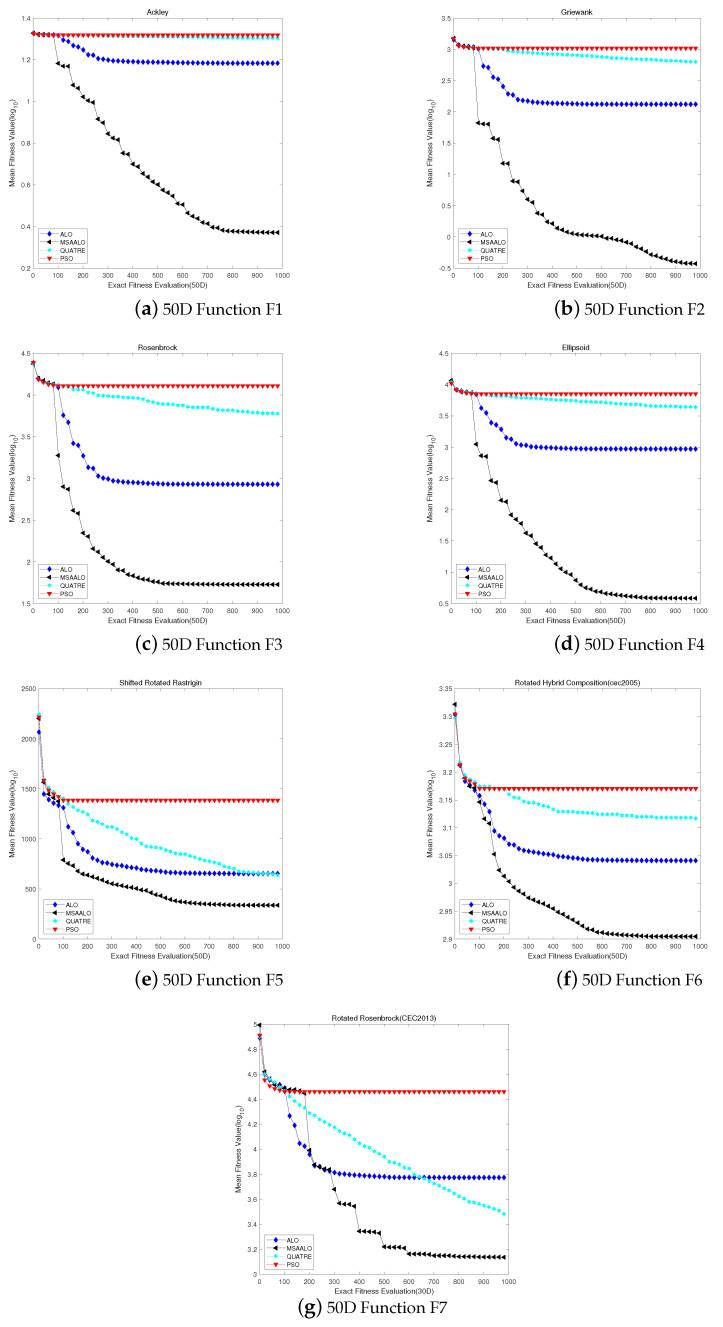
Convergence profiles of algorithms MSAALO, PSO, ALO and QUATRE on 50D with 1000 expensive fitness evaluations.

For the 50-dimensional problem, Figure 2 shows the convergence curves of four comparison algorithms. For the convergence curves on F1 and F2, MSAALO initiates to converge around 900 generations; ALO starts to converge around 300 generations; PSO and QUATRE begin to perform convergence around 200 generations. The convergence curves prove that MSAALO is stronger than the other three algorithms in the global search for the F1 and F2 benchmark functions. ALO, PSO and QUATRE algorithms encounter the problem of local convergence earlier. For F3–F4 and F6 benchmark functions, MSAALO converges around 600 generations; ALO starts to converge around 300 generations; PSO starts to converge around 200 generations; QUATRE begins to converge at a later stage, but QUATRE is not as good at global search as MSSALO. Compared to PSO and ALO, MSAALO initiates to converge in the late evaluations. These demonstrate that MSSALO is more robust than the other three algorithms in capturing global profiles. On the F5 benchmark function, QUATRE keeps exploring and converges at 800 generations, while MSAALO converges at 600 generations, but the graphs show that MSAALO has a more vital search capability than QUATRE. Although MSAALO converges in 600 generations, the image shows that MSAALO has a more robust search capability. Compared with the 30-dimensional pictures, MSAALO has better exploitation and exploration ability, proving that MSAALO effectively solves expensive high-dimensional problems.

### 4.3. Experimental Analysis on 100-Dimensional Problems

**Figure 3:**
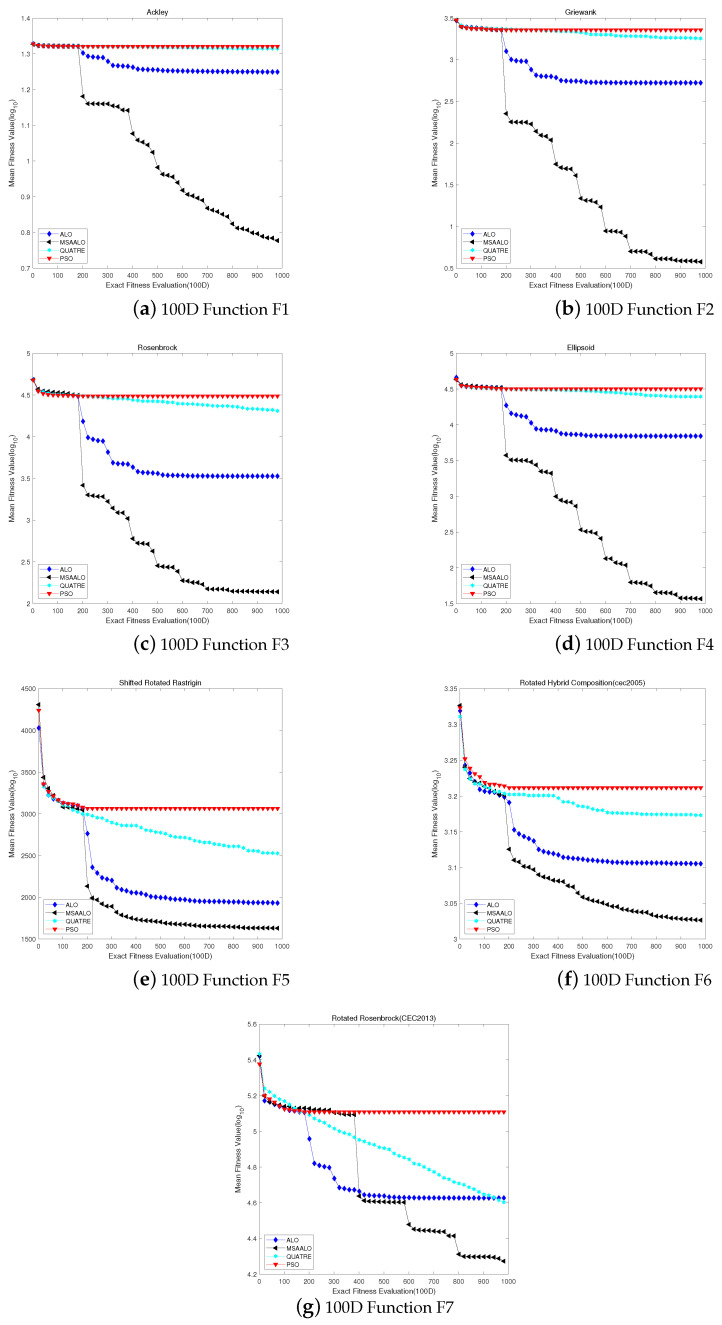
Convergence profiles of algorithms MSAALO, PSO, ALO and QUATRE on 100D with 1000 expensive fitness evaluations.

In this section, we compared MSAALO with ALO, PSO and QUATRE in the 100 ultrahigh dimension benchmark functions. Table 4 shows the experimental results of the working process in the 100-dimensional benchmark function. Figure 3 shows the convergence curve of these comparison algorithms. The analytical results of these benchmarks of MSAALO and the three other algorithms for comparison in the 100 dimensions are displaced in the Table 4. Some evident conclusions can be obtained from this table. MSAALO is better than the other three algorithms for comparison based on the statistics in all these benchmark functions. We infer some efficient summary from Ackley, Griewank and Ellipsoid. The average values of the results acquired by MSAALO are close to the true optima. For the other functions, these algorithms cannot locate efficient areas. The information presented in these figures and tables shows that MSAALO efficiently solves expensive optimization problems. We see from these figures that MSAALO achieves the optimal value earlier than the other algorithms.

For Ackley and Griewank test functions, MSAALO converges around 1000 evaluations; ALO converges around 300 evaluations; PSO and QUATRE start to perform convergence around 100 evaluations. ALO, PSO and QUATRE algorithms encountered the problem of local convergence very early. For Ellipsoid and Rotated Rosenbrock test functions, MSSALO converges around 900 evaluations; ALO converges around 300 evaluations; PSO converges around 100 evaluations; QUATRE has better exploring ability than the convergence curve. We can see that the MSAALO algorithm has a better global ability for the best solutions. The convergence curves of Rosenbrock show that MSAALO has a better exploration ability and achieves a good balance between exploitation and exploration. In the Rotated Hybrid Composition (F6) benchmark function, MSAALO converges in 900 evaluations; ALO converges in 400 evaluations; QUATRE converges in 600 evaluations; PSO converges in 200 evaluations. The other three algorithms converge later out of the Shifted Rotated Rastrigin benchmark function. Compared with the 50-dimensional problems, MSSALO has a more vital ability to capture the global best solution, demonstrating that MSSALO is prevalent in solving high-dimensional problems.

### 4.4. WSN Coverage Optimization Experiment Simulation

In this paper, MSAALO is employed to address the sensor coverage problem of WSN. MSAALO optimizes the positions of sensor nodes to reach the maximum coverage area. In order to verify the validity of this algorithm for optimizing the coverage area of WSN, this study compared MSAALO with PSO, ALO, QUATRE in this application. First, we tested the coverage rate in a different amount of sensor nodes with the same radius (5 m). The amount of sensors vary from 30 to 55. Secondly, we validated the effectiveness with different sensor radius and a fixed number of sensor nodes (30). The variation range of radius is from 5 m to 10 m. The ants’ size is equal to 50, and the round time is 10. The four algorithms independently run 30 times to compare. From the coverage rates of 30 times, this study computes the average rates to analyze. This research included a simulation experiment in the area of plane of 50 m × 50 m in the three-dimensional terrain.

Table 5 represents the comparing results of coverage rate using the different number of sensor nodes with identical radius. Table 6 shows the comparing results of coverage rate using different sensor detecting radius with the same amount of sensor nodes (30). From these data, this article analyze that MSAALO has more advantages to address the complication situation as the number and radius of sensor nodes increase. The simulation results based on different radii and nodes show that MSAALO improves the average coverage by 2.122% and 1.718%, respectively.

## 5. Conclusions

In order to improve the sampling technology of the surrogate models and the convergence performance of ALO, this paper proposes a Mahalanobis Sampling Surrogate-Assisted Ant Lion Optimization (MSAALO) algorithm. There are three layers: the local surrogate, Mahalanobis sampling surrogate model and global model. Together, these triple layers shape the holistic framework of MSAALO. By using RBFN, the whole model is trained. The algorithm uses Mahalanobis distance to obtain some samples in the Mahalanobis sampling surrogate model. Then, the algorithm uses these samples to form a surrogate model. The global surrogate model has the ability of global fitting of smooth functions. The local surrogate model mainly acquires the function model around the currently best ant to enhance the search ability of the algorithm. This work uses seven benchmark functions to test to verify the effectiveness in dealing with high-dimensional time-consuming problems. The validation data in 30 dimensions, 50 dimensions, and 100 dimensions demonstrates that MSAALO is competitive. In order to verify the effect in practical application, this study simulates the 3D deployment effect of Wireless Sensor Networks. MSAALO also has a significant number of spaces to improve. Future work could enhance the diversity of selected samples. Additionally, in order to improve the surrogate’s ability to acquire the global landscape more precisely of the real problem, we should choose various models. Future work must incorporate refining the model or algorithm to solve real multi-objective large-scale world problems, as well. 

## Figures and Tables

**Table 1:** Benchmark.

Benchmark Function	Name	Characteristics	Global Optimal
F1	Ackley	Multimodal	0
F2	Griewank	Multimodal	0
F3	Rosenbrock	Multimodal with narrow valley	0
F4	Ellipsoid	Unimodal	0
F5	Shifted rotate Rastrigin(F10 in [53])	Very complicated multimodal	−330
F6	Rotated Hybrid composition function(F16 in [53])	Very complicated multimodal	120
F7	Rotated Rosenbrock’s Function(F6 in [55])	Multimodal	−300

**Table 2:** Statistical results of the proposed algorithm MSAALO and the comparison algorithm in the 30D benchmark problems.

Function	Method	Best	Worst	Mean	Std.
F1 Ackley	MSAALO	0.123326744	3.13585031	**1.43972806**	0.849227975
	ALO	12.10555809	15.6481591	14.19208313(+)	0.9459971
	PSO	20.15494482	20.82542968	20.59548416(+)	0.172703454
	QUATRE	20.31594928	20.8686191	20.62503928(+)	0.163136984
F2 Griewank	MSAALO	0.025057236	0.348212147	**0.20459889**	0.105483642
	ALO	34.50123904	85.11353248	61.12324826(+)	13.39587586
	PSO	399.9440594	629.2822719	539.9281838(+)	58.72504985
	QUATRE	1430.698577	2134.531336	1793.335464(+)	181.220765
F3 ROSENBROCK	MSAALO	27.33130867	31.2019712	**28.919217**	0.911458613
	ALO	212.0563715	472.0551911	329.7765029(+)	73.50371706
	PSO	3612.815328	8177.474992	5768.083768(+)	1056.556912
	QUATRE	13816.70038	25796.98324	20242.16778(+)	3580.971155
F4 Ellipsoid	MSAALO	0.064639725	1.745649711	**0.76747429**	0.542000353
	ALO	137.5505289	316.3088353	236.7281153(+)	47.19823742
	PSO	1869.607222	2942.181818	2298.538358(+)	266.6575441
	QUATRE	18719.26637	29801.73299	24745.30049(+)	3096.902059
F5 Shifted Rotated Rastrigin’s	MSAALO	−177.3906961	41.14829764	**−97.86071**	62.85805275
	ALO	−28.50723525	190.6024357	121.7342326(+)	61.58425884
	PSO	349.4033779	684.5691941	560.3924598(+)	97.33286262
	QUATRE	2210.59487	2905.192736	2527.153953(+)	161.6924755
F6 Rotated Hybrid Composition	MSAALO	487.8856464	883.2270767	**657.345355**	91.4233247
	ALO	680.0437144	1185.978105	931.6602035(+)	144.3053267
	PSO	1012.886072	1529.59108	1240.718675(+)	146.1381248
	QUATRE	993.5244617	1254.09476	1126.998238(+)	69.97195687
F7 Rotated Rosenbrock’s Function (cec2013)	MSAALO	305.9999456	968.5415869	**497.931191**	166.910778
	ALO	2516.102941	8820.339926	5375.970745(+)	1790.558688
	PSO	13511.26453	28774.73239	21609.56369(+)	4160.450511
	QUATRE	371.1491082	1080.859897	580.0806404(+)	190.9446216

**Table 3:** Statistical results of the proposed algorithm MSAALO and the comparison algorithm in the 50D benchmark problems.

Function	Method	Best	Worst	Mean	Std.
F1 Ackley	MSAALO	0.940415879	5.167946126	**2.3517038**	1.251470448
	ALO	13.82370384	16.68453778	15.25804536(+)	0.796298412
	PSO	20.54068171	20.9449642	20.81156212(+)	0.095431374
	QUATRE	19.56914323	20.50332195	20.09924447(+)	0.22904872
F2 Griewank	MSAALO	0.104616818	0.926048755	**0.37396578**	0.216959396
	ALO	105.2416856	174.1706141	132.4385061(+)	21.29345499
	PSO	941.2522423	1143.06337	1043.479008(+)	63.8714882
	QUATRE	511.6977831	739.5520411	627.2609005(+)	68.17036889
F3 ROSENBROCK	MSAALO	48.49067593	75.15137415	**53.4668016**	8.024656915
	ALO	592.1867282	1107.620799	851.1901101(+)	129.4974263
	PSO	9478.242386	15087.31686	12884.91921(+)	1425.141242
	QUATRE	4668.454943	7782.74757	5958.326781(+)	803.8444471
F4 Ellipsoid	MSAALO	0.183039002	15.10150372	**3.84620633**	3.659057369
	ALO	688.2929038	1333.181207	935.4220777(+)	173.2759225
	PSO	6395.877995	7872.311698	7117.623372(+)	385.1067671
	QUATRE	3115.474344	5157.058137	4328.406839(+)	475.7695647
F5 Shifted Rotated Rastrigin’s	MSAALO	160.0359335	626.0513082	**337.473869**	126.853667
	ALO	495.0934545	757.8380202	653.8728804 (+)	77.2052377
	PSO	1194.449781	1551.690511	1386.945263(+)	98.67065573
	QUATRE	499.8560298	731.0150509	629.5159052(+)	74.64785504
F6 Rotated Hybrid Composition	MSAALO	558.4800991	1040.265314	**802.894535**	161.6001346
	ALO	951.0065564	1216.362594	1099.303637(+)	89.67953714
	PSO	1360.985177	1601.730509	1481.080647(+)	70.85044609
	QUATRE	643.4407881	991.0314188	805.936404(≈)	90.57789709
F7 Rotated Rosenbrock’s Function (cec2013)	MSAALO	741.5338717	2449.577822	**1368.97891**	395.6821817
	ALO	3968.852388	9349.459488	5934.070009(+)	1408.381766
	PSO	20661.10026	37463.82879	28844.48817(+)	4722.00103
	QUATRE	1622.13098	4279.212564	2891.371949 (+)	640.6297767

**Table 4:** Statistical results of the proposed algorithm MSAALO and the comparison algorithm in the 100D benchmark problems.

Function	Method	Best	Worst	Mean	Std.
F1 Ackley	MSAALO	4.091671039	8.774707511	**5.93663355**	1.261160452
	ALO	17.21809925	18.35013969	17.75140425(+)	0.252252221
	PSO	20.77643095	21.02382447	20.92523434(+)	0.056834438
	QUATRE	20.31594928	20.8686191	20.62503928(+)	0.163136984
F2 Griewank	MSAALO	1.851830569	6.001070319	**3.76704066**	1.069549415
	ALO	464.5274265	583.0803864	528.7988196(+)	39.70428045
	PSO	1943.472754	2498.344413	2276.611987(+)	107.2391004
	QUATRE	1430.698577	2134.531336	1793.335464(+)	181.220765
F3 ROSENBROCK	MSAALO	112.0862083	211.8498645	**138.034242**	26.45273892
	ALO	2410.659629	4175.374741	3366.246959(+)	441.5179758
	PSO	27037.12808	34123.8097	30717.76802(+)	1815.48804
	QUATRE	13816.70038	25796.98324	20242.16778(+)	3580.971155
F4 Ellipsoid	MSAALO	22.89635178	73.41179558	**35.8898755**	14.07490228
	ALO	5696.304718	8042.695737	6953.813749(+)	662.0899404
	PSO	28389.28814	33571.10473	31854.291(+)	1220.854732
	QUATRE	18719.26637	29801.73299	24745.30049(+)	3096.902059
F5 Shifted Rotated Rastrigin’s	MSAALO	1489.06262	1799.211178	**1629.79298**	95.1636403
	ALO	1655.179877	2148.085759	1926.3667(+)	124.7194161
	PSO	2857.938669	3291.183242	3066.387997(+)	113.9989264
	QUATRE	2210.59487	2905.192736	2527.153953(+)	161.6924755
F6 Rotated Hybrid Composition	MSAALO	848.527674	1255.817093	**1062.61981**	133.5560952
	ALO	1122.289105	1417.145008	1274.688185(+)	80.250579
	PSO	1512.055362	1740.348203	1627.7907(+)	63.57016225
	QUATRE	993.5244617	1254.09476	1126.998238(+)	69.97195687
F7 Rotated Rosenbrock’s Function (cec2013)	MSAALO	10046.25135	22562.53428	**16436.0136**	3689.60855
	ALO	35859.80184	54097.03908	42368.11593(+)	5130.810022
	PSO	92916.94975	152443.4916	128356.6319(+)	14760.00506
	QUATRE	28101.80237	49374.69153	38759.84115(+)	5441.075378

**Table 5:** Comparing results with different numbers of nodes.

Num	MSAALO	ALO	PSO	QUATRE
30	47.56%	36.22%	**47.72%**	41.16%
35	**51.74%**	40.61%	51.08%	45.48%
40	**59.45%**	44.83%	56.68%	50.56%
45	**62.14%**	48.96%	59.12%	55.00%
50	**66.87%**	52.54%	64.84%	59.28%
55	**69.73%**	55.88%	67.60%	60.92%

**Table 6:** Comparing results with different radii.

Radius	MSAALO	ALO	PSO	QUATRE
5m	47.56%	36.22%	**47.72%**	41.16%
6m	**61.96%**	47.15%	60.88%	55.92%
7m	**75.45%**	57.58%	74.28%	66.44%
8m	**86.14%**	66.48%	84.28%	76.72%
9m	**92.59%**	69.88%	90.60%	83.48%
10m	**96.93%**	79.24%	94.44%	89.96%

## Data Availability

Not applicable.
